# Oscillatory Brain Activity Reveals Linguistic Prints in the Quantity Code

**DOI:** 10.1371/journal.pone.0121434

**Published:** 2015-04-15

**Authors:** Elena Salillas, Paulo Barraza, Manuel Carreiras

**Affiliations:** 1 BCBL. Basque Center on Cognition, Brain and Language. Donostia, Spain; 2 CIAE. Centro de Investigación Avanzada en Educación, Universidad de Chile, Santiago, Chile; 3 IKERBASQUE, Basque Foundation for Science, Bilbao, Spain; University of Hyderabad, INDIA

## Abstract

Number representations change through education, although it is currently unclear whether and how language could impact the magnitude representation that we share with other species. The most prominent view is that language does not play any role in modulating the core numeric representation involved in the contrast of quantities. Nevertheless, possible cultural hints on the numerical magnitude representation are currently on discussion focus. In fact, the acquisition of number words provides linguistic input that the quantity system may not ignore. Bilingualism offers a window to the study of this question, especially in bilinguals where the two number wording systems imply also two different numerical systems, such as in Basque-Spanish bilinguals. The present study evidences linguistic prints in the core number representational system through the analysis of EEG oscillatory activity during a simple number comparison task. Gamma band synchronization appears when Basque-Spanish bilinguals compare pairs of Arabic numbers linked through the Basque base-20 wording system, but it does not if the pairs are related through the base-10 system. Crucially, this gamma activity, originated in a left fronto-parietal network, only appears in bilinguals who learned math in Basque and not in equivalent proficiency bilinguals who learned math in Spanish. Thus, this neural index reflected in gamma band synchrony appears to be triggered by early learning experience with the base-20 numerical associations in Basque number words.

## Introduction

Does language learning modify our numerical system? Basic magnitude processing implies the specialization of the quantity code with an essential role of the intraparietal sulcus, but also the orchestration of temporo-occipital, left inferior parietal or frontal cortex in the acquisition of numerical symbols and for arithmetic learning [1–5] Verbal names for quantity are learned before the Arabic system. It seems thus plausible that developmental changes in the quantity code could be shaped by the syntactic structures imposed by the numeric wording system and, in turn, that an evolved representation for quantity incorporates linguistic traces.

A test for possible long-lasting associations between particular number wording systems and quantity was possible through the selection of proficient, balanced Spanish-Basque bilinguals, who had learned math either in Basque (LL_B_^math^ group) or in Spanish (LL_S_^math^ group). In the case of Basque-Spanish bilingualism, number words from the two languages are not just two codes for naming a certain quantity, but imply two different systems for numeric organization: vigesimal (base 20) vs. decimal (base 10) systems. Here, we measured the implicit effects of these two different numerical systems on brain oscillations during Arabic digit comparison. Number comparison is generally taken as a task that taps into an abstract analogue magnitude representation [6,7]. Thus, the comparison of Arabic digits, even if calling to a symbol-to-quantity neural map, should not be interfered by other possible (verbal) formats. Therefore, if number wording effects are visible when comparing Arabic digits, it would imply that a numeric representation with linguistic components exists and has been accessed.

This neurodynamic relationship between language and the representation of numerical magnitude was evaluated here by measuring the electroencephalography (EEG) response while bilingual participants performed a numeric size comparison task between pairs of Arabic digits. A subsequent analysis of large-scale neural synchronization was carried out by computing the phase coupling [8,9] between all pairs of electrodes in the EEG. If the language system modulates the core number representation, qualitative differences in the time course of the frequency bands in phase synchronization to digit pairs with a vigesimal vs. decimal relationship are expected. Moreover, the analysis of the electrodes sites with coherent phase at similar frequencies will help us to outline possible different networks in the processing of vigesimal links. Crucially, by analyzing this activity in groups with a different learning experience in the vigesimal wording system a possible specific early mapping into quantity can be explored: base 20 traces originated during early acquisition of number words and number concepts would appear only for those participants whose LL^math^ includes that base 20 system in its number words (i.e. the LL_B_^math^ group).

## Material and Methods

### Participants

Eighteen equally proficient Spanish-Basque bilinguals participated in this study (from Salillas and Carreiras, 2014 [10]) The average age of acquisition of L2 Basque was 1.5 years (minimum 0 years, maximum 6 years). Nine participants reported learning math in Spanish (LL_S_^math^ group) and nine reported learning math in Basque (LL_B_^math^ group). Aside from LL^math^, the groups were equivalent in proficiency: for the LL_S_^math^ group_,_ proficiency in Spanish, as measured by the Boston Naming Test (BNT) [11] was 54.8 (2.52), and proficiency in Basque was 48.8 (3.56) (t = 2.29; p = 0.05); for the LL_B_^math^ group, BNT scores were 53.4 (2) and 50.2 (3.63) respectively (t = 5.6; *p* < 0.001). Thus, both groups were slightly more proficient in Spanish and were equivalent in relative proficiency (t = 1.96, p = 0.09). On average, the LL_B_^math^ group self-reported using Basque 39% of the time and Spanish 61% of the time. The LL_S_^math^ group used Basque 44% of the time and Spanish 56% of the time.

### Ethics statement

This study was approved by the Ethical Committee for Clinical Research within the Guipuzcoan public health sector (CEIC), which is the pertinent external committee for the assessment of studies involving human subjects in our region. The study was approved attending to the provided information: procedures to recruit participants (e.g. number of participants, inclusion/exclusion criteria, the risks and benefits for the participants), informed consent procedures that were implemented, copies of the information sheets and consent forms. Participants gave written informed consent. The application of all relevant European and national (Real Decreto 223/2004 Feb-6) was assessed (i.e. Declaration of Helsinki). This assessment was requested by the FP7-PEOPLE-2010-IEF funding body, which also assessed all the relevant ethical documentation and procedures.

### Stimuli

A subset of the stimuli included in Salillas and Carreiras (2014) [10] ERP study was analyzed here. This subset comprised those pairs of digits in which the target digit was exactly the same number for all conditions. Pairs of digits were constructed according to the verbal forms of Spanish and Basque numbers: Common pairs and Basque pairs. For the Common pairs, 36 pairs were constructed according to the decimal system, which is common to the verbal form in Basque and Spanish. For example, the expression for the verbal form of “forty-six” is the same in both Spanish (“cuarenta y seis”) and Euskera (“Berrogeita sei“). This is the case for all digits that contain an even decade (e.g., 45, 67, 89, and so on). Each pair to be compared consisted of the whole number (e.g., 45) (the first number), and the decade included in the number word (e.g., 40) (the second number). The Basque pairs comprised 36 pairs constructed according to the Basque expression of numbers, based on the vigesimal system. The first number was a two-digit number with an odd decade (e.g., 55, 76, 99, and so on). The second digit implied the use of the closest even decade, which, according to the vigesimal system, is part of the number word. For example, 56 is “cincuenta y seis” in Spanish (fifty-six), but is “berrogeita hamasei” (forty and sixteen) in Basque.

The hypothesis and relevant contrasts were thus based on a comparison of responses to the two types of pairs across groups, with identical items used for each group of participants and identical target numbers for both Basque and Common pairs.

### Procedure

For all the 72 experimental pairs, the largest number (e.g., 68) was presented first. In order to avoid the inference of any rule, 36 fillers were presented in the same order (large then small), and a pool of 108 fillers were presented in the opposite order (small then large). These filler pairs did not follow any verbal form relationship and had random numerical distance between the first and second number.

**Fig 1:**
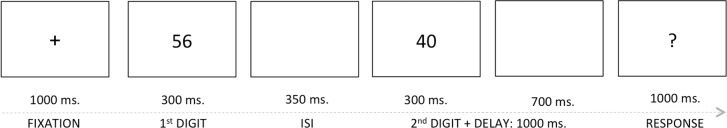
Example of one trial. Each trial consisted of a fixation point that appeared for 1000 ms, a first number that lasted 300 ms. followed by a blank screen for 350 ms. and finally a second number that appeared for 300 ms. After 700 ms a delayed response was requested.

Each trial consisted of a fixation point that appeared for 1000 ms followed by a first number that remained on the screen for 300 ms. This first number was followed by an ISI of 350 ms in which a blank screen was presented. The second number then appeared for 300 ms. The appearance of the second number was the onset trigger for EEG analyses. After a blank screen had been shown for 700 ms, a question mark signaled the request of a delayed response (see Fig 1 for a display of a trial). A delayed response was introduced in the task in order to avoid motor preparation interferences in the EEG response, analyzed after the second digit. The task of each participant was to decide whether the second number was larger or smaller than the first number by pressing one of two buttons.

### EEG recording

EEG was recorded from 27 scalp electrodes embedded in an Easy-Cap in a 10–10 system array, and was referenced online to the left mastoid. Six additional electrodes were used to record blinks (from below the eye) and horizontal eye movements (placed at the outer canthi), using the left mastoid processes as a reference. Electrode impedances were maintained below 5 kOhms. The EEG was amplified with Brain Amp amplifiers, with the band pass set from 0.01 to 100 Hz, and sampled at a rate of 500 Hz. The output of the bioamplifiers was fed into a 32-channel, 12-bit analogue-to-digital converter, which was located inside a PC computer. Presentation software was used to present the visual stimuli and record the behavioral responses. Brain Vision Recorder software was used to deliver the event and timing codes to the data acquisition PC, so that the epochs of EEG activity corresponded precisely to the events of interest. Trials with artifacts due to eye movements, excessive muscle activity, or amplifier blockage were eliminated semi-automatically offline, in order to restrict them to the analyzed segment. Maximal allowed absolute difference after time = 0 ms. in each segment was kept to 150 μV. Maximal and minimal allowed amplitude was +90 μV and -90 μV respectively. And lowest activity (max—min) was kept to 0.5 μV. The average number of trials by condition was: LL_S_^math^ 30.5 (sd 3.7) for Common pairs and 29.8 (sd 3.4) for Basque pairs; LL_B_^math^ 31.3 (sd 3.8) for Common pairs and 28.4 (sd 3.8) for Basque pairs.

### Data analysis

The raw EEG signal was first segmented into a series of epochs lasting 2350 ms including 1350 ms preceding the second digit. Electrodes placed near the eyes were excluded from the analysis in order to avoid biological artifacts. The continuous 50 Hz (AC) component was filtered in each epoch using a zero-phase filter that keeps the biological 50 Hz signal. Then, the filtered signal was analyzed with a sliding-window fast Fourier transform (window length, 128 ms; step, 10 ms), in all trials and subjects for each condition. Using this process, we obtained the signal phase for frequencies between 1 and 70 Hz, with 1 Hz frequency resolution. Phase information was then used to compute a time-varying phase-locking value (PLV), which is an index of neural synchrony [8, 9,12] This method involves computing the phase difference in a time window for an electrode pair, and assessing the stability of such a phase difference for all trials. If Φi and Φj are unitary vectors representing the phase of EEG signals in electrodes i and j, phase differences are represented by unitary vectors obtained using the following equation:

$$\Phi_{\text{ij}}=\Phi_{\text{i}}\text{conj }(\Phi_{\text{j}})$$

The PLV is the length of the vector resulting from the vector sum of difference vectors for the trials (with the sum operating throughout all of the trials), where N is the number of trials:

$${\text{PLV}}_{\text{ij}}=\text{ abs }(\Sigma \Phi_{\text{ij}})/\text{ N}$$

The PLV index ranges from 0 to 1, with a value of 1 indicating perfect synchronization (i.e., phase difference that is perfectly constant throughout the trials), and a value of 0 representing the total absence of synchrony (i.e., phase differences are random). Phase synchronization across an entire trial for each frequency bin was normalized to a 400 ms baseline preceding TN second digit onset. The normalized signal (S_N_) was obtained by subtracting the average activity of the baseline (μ) from the filtered signal (S) divided by the standard deviation of the baseline (σ), in a frequency-by-frequency manner:

$${\text{S}}_{\text{N}}=\left(\text{S - μ}\right)/\sigma$$

### Statistical analysis

Because we were interested in long-range coordination of neural activity, we included all electrodes in the calculation to produce a global index of synchronization across a large frequency range. The statistical analysis of the phase synchrony was performed on time-frequency charts resulting from averaging the electrophysiological responses of all electrodes pairs, from 0ms to 700ms after target number onset. This resulted in a grand average time-frequency and a phase synchrony chart per experimental condition per subject. Then, those charts were grouped by condition and analyzed by means of a permutation test (α = 0.05) in search of time-frequency windows showing significant effects [13]. Subsequently, the significant time-frequency windows were analyzed with a between-subject ANOVA. The α level was set at 0.05 for all tests and, when necessary, we applied Greenhouse-Geisser correction.

For the topographical analysis of phase synchrony we controlled for the statistical effects of multiple comparisons by choosing a very conservative significance threshold (p < 0.00006). This threshold was set on the basis of the distribution of synchrony values during the baseline. The threshold was chosen such that the number of cases larger than the threshold divided by the total number of cases was equal to p = 0.00006. By choosing this significance level, one line per analysis window could be explained by chance, given the fact that there were 27 electrodes with 351 possible combinations (27x26/2 = 351) (For similar method, see [14,15, 16]).

We performed the analysis of EEG phase synchrony with Matlab 7.0.4 (Mathworks, Inc) using algorithms developed by Dr. Eugenio Rodriguez and others [8].

## Results

### Phase synchrony

**Fig 2:**
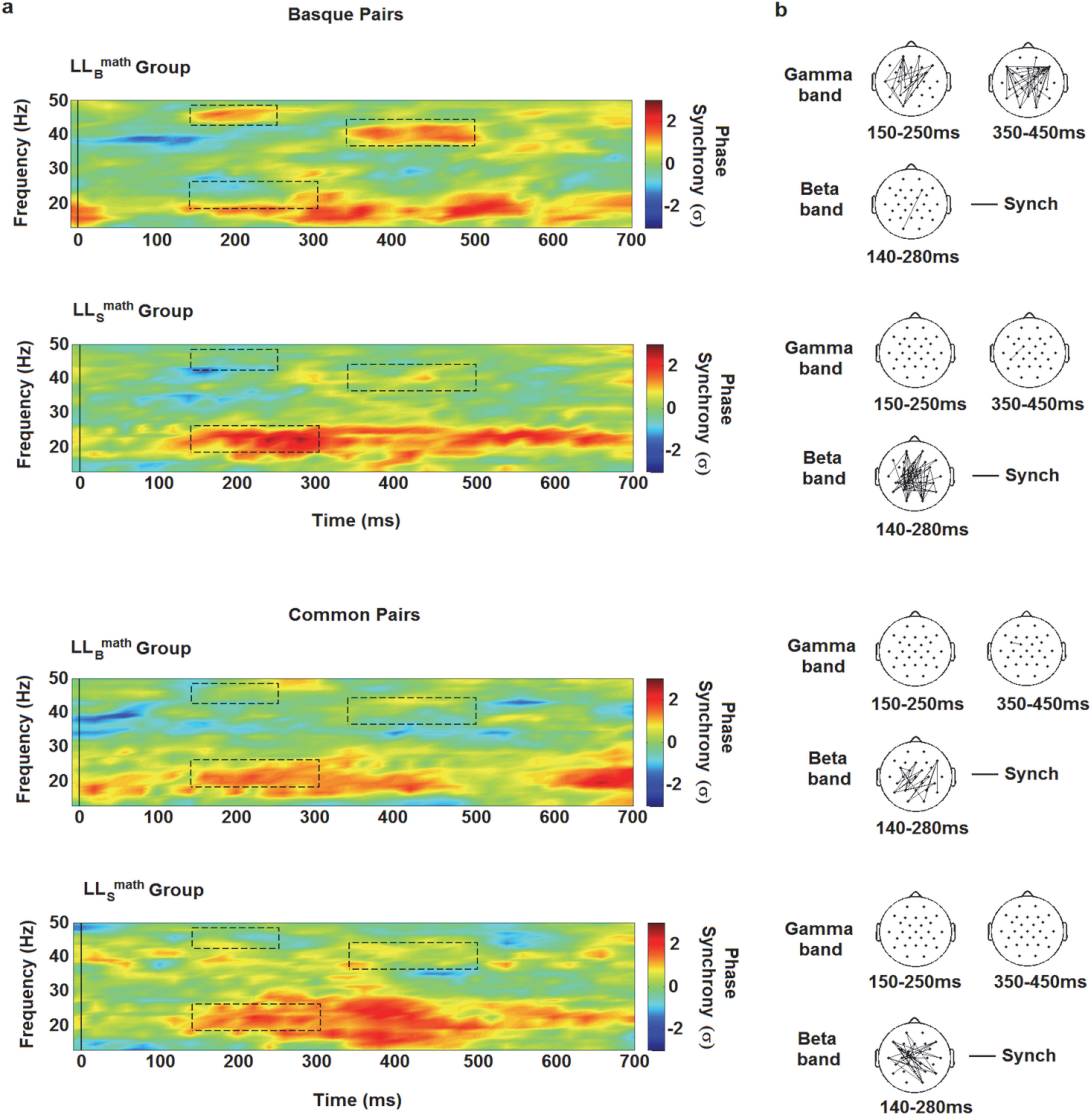
Phase Synchrony and its distribution over the scalp for each group and experimental condition. a) Frequency range and time are respectively indicated in the y and x-axis of the maps. Color bars at the right side of the maps show the mean phase-locking value (in standard deviation units) between all electrodes pairs. Vertical lines indicate the TN second digit onset. The black segment rectangles delimit time windows showing significant differences between conditions for the LL_B_^math^ and LL_S_^math^ groups (*p* < 0.05). b) Spatial distribution of phase synchrony for each significant time-frequency windows. The points represent the location of the electrodes over the scalp. Frequency bands are indicated at the left of each row. Time windows are indicated at the bottom of each head. Black lines connect pairs of electrodes displaying significant synchronization (*p* < 0.00006).

Results are shown in Fig 2A. For the Basque pairs condition, we found that mean Gamma phase synchrony over all electrode pairs was significantly higher for LL_B_^math^ group than LL_S_^math^ group from 150 ms to 250 ms (frequency: 43–46 Hz, F_1,16_ = 5.387, *p* = 0.034, *η* ^*2*^ = .252) and from 350 ms to 450 ms (frequency: 38–42 Hz, F_1,16_ = 6.489, *p* = 0.022, *η* ^*2*^ = .289) after TN second digit onset, whereas Beta phase synchrony was significantly higher for LL_S_^math^ group than LL_B_^math^ group from 140 ms to 280 ms (frequency: 22–26 Hz, F_1,16_ = 10.798, *p* = 0.005, *η* ^*2*^ = .403) after TN second digit onset. For the Common pairs condition, we not observed significant differences in these time-frequency windows between the LL_B_^math^ group and LL_S_^math^ group.

The spatial distribution of phase synchrony over the scalp for each experimental condition is depicted in Fig 2B. In the case of the LL_B_^math^ group, we observed two significant increase of Gamma phase synchronization during Basque pairs condition. The first one among fronto-parietal sites mostly over the left side of the scalp, from 150 ms to 250 ms after TN second digit onset, and the second between bilateral frontal and parietal sites, from 350 ms to 450 ms after TN second digit onset. In the same condition, the LL_S_^math^ group showed a strong increase of Beta phase synchronization mainly among occipital-parietal site, from 140 ms to 280 ms after TN second digit onset. During Common pairs condition, we observed an increase in Beta phase synchrony, but not Gamma, in both groups, among posterior sites mostly over the left side of the scalp, from 140 ms to 280 ms after TN second digit onset.

## Discussion

The results reveal that language shapes the magnitude representation that we share with other species. It has previously been shown that early math learning shapes memory networks for arithmetic [17]. We have recently shown that early math learning enters the quantity code [10]. We demonstrated that this early learning impacts a certain magnitude marker such as the distance effect [18]. Here we further show this most fundamental language learning impact on basic numeric representations. Taking advantage of the difference in the Basque-Spanish use of number naming systems, the results of the current study crucially show that the specific way in which we name numbers penetrates our number representational system.

The data show clear differences between the two groups in terms of the frequency bands and phase synchrony patterns associated with comparisons related to the base 20 system. The differences, which appear around 200 ms after participants start the comparison process, suggest gamma band activation likely based on a left fronto-parietal network. It is the activity of that fronto-parietal network in gamma phase synchrony what seems to be specific to the Basque pairs for the LL_B_^math^ group. The early synchronized fronto-parietal network may reflect the maintenance of verbal to quantity links, as suggested by recent proposals [3, 5, 17]. In addition, a second synchronization in gamma band occurs in a similar parieto-frontal network but this time, bilaterally. This two-phase gamma band activity suggests the spread of the coherent activity to the opposite hemisphere, in a later moment. Thus although an initial language-related left lateralized network starts the essential quantity processing other non-linguistic processes might play a role in a second moment during the comparison of base-20 pairs by this group. In turn, the performed analysis nicely describes how this two-phase processing unfolds over time in an increasingly extended network.

All the rest of conditions showed similar beta activity in bilateral central-posterior networks. This latter network was common to both groups when early acquired verbal links were not used (i.e. for both pairs in the LL_S_^math^ and for Common pairs in LL_B_^math^), and is thus posited to reflect non-linguistic, quantity-related processes. Note that the base 10 system is not only a naming system, but a system in which math is generally structured (i.e., decimal metric system). Therefore, the neural integration of the base 10 system does not need to be linguistic in origin. In fact, the base 10 system has a route in the widespread use of fingers for counting. Base 10 is used for the place coding in multi-digit numbers. In turn, base 10 structure is a feature of a particular representational system rather than a fundamental mathematical fact. Nevertheless, it is a feature that is incorporated into many of the algorithms that children learn for arithmetic performance [19].

It is the claim of all theoretical approaches that a verbal code is not needed when magnitude is contrasted using Arabic digits [5–7, 20]. Even most interactive models [21] assume that a comparison task without verbal input does not activate verbal processes [22]. There is consensus that this task operates via the use of the analogue magnitude code. Therefore, the finding of a number word related effect associated with this task suggests linguistic traces within this code in the form of networks established through learning. Decimal relations have been proposed as being crucial in the semantics of numbers [20], but in the absence of a verbal response this proposal did not predict an effect of verbal syntax during the processing of Arabic numbers. Additionally, there is a current focus on how symbols get linked to the abstract code as a fundamental achievement in mathematic functions, and as a key for successful arithmetic and complex calculation [5,23,24]. By taking the advantage of the coexistence of two ways of naming numbers in Basque-Spanish bilinguals, our data suggest that number words have, in fact, left a permanent signature that is closely linked to the quantity code.

In neurophysiological terms, the reciprocal relationship between the number system and number words could imply the integration—dynamic and transient—of neural networks widely distributed across the brain. It has been proposed that the integration of brain activity is the result of transient synchronization of neural activity [9]. Neural synchronization implies that the brain sites that enter into synchrony are functionally related to each other, and therefore implicated in processes relevant to the performance of cognitive tasks. The present data suggest that long distance integration in the gamma band [9, 25–28] occurs between frontal and centro-parietal areas when the base 20 system is activated. A representational network may have originated in early learning, which appears to integrate in gamma band even during simple digit comparison without a call to a verbal channel. The onset of this synchronization (around 200 ms) coincides with the timing reported by other studies [29–31] that focused on number semantic effects in comparison tasks. This timing suggests that the reported effects occur at the stage when semantics are being accessed. In addition, synchronization in beta band suggests that processes/representations common to non-linguistic (i.e. base 10) processes, needless of integration in gamma supported the processing of the other conditions. Again, functional networks for the processing of decimal relations which originated in other sources than number words and which have even been proposed as essential for numerical semantics, contrast with the integrated and more extended functional networks for the linguistic information carried by vigesimal pairs. In turn, the present data strongly suggest that language links to quantity very early in life when words and quantity are first combined.
