# Correction: Oscillatory brain activity reveals linguistic prints in the quantity code

**DOI:** 10.1371/journal.pone.0130236

**Published:** 2015-06-03

**Authors:** Elena Salillas, Paulo Barraza, Manuel Carreiras

The following information is missing from the Funding section: PB was supported by the program CONICYT PAI/Academia (grant 79130005).
